# NADPH biosensor-based identification of an alcohol dehydrogenase variant with improved catalytic properties caused by a single charge reversal at the protein surface

**DOI:** 10.1186/s13568-020-0946-7

**Published:** 2020-01-18

**Authors:** Alina Spielmann, Yannik Brack, Hugo van Beek, Lion Flachbart, Lea Sundermeyer, Meike Baumgart, Michael Bott

**Affiliations:** IBG-1: Biotechnology, Institute of Bio- and Geosciences, Forschungszentrum Jülich, Jülich, Germany

**Keywords:** NADPH biosensor, *Lactobacillus brevis*, NADPH-dependent alcohol dehydrogenase, Enzyme optimization, Fluorescence-activated cell sorting, Random mutagenesis

## Abstract

Alcohol dehydrogenases (ADHs) are used in reductive biotransformations for the production of valuable chiral alcohols. In this study, we used a high-throughput screening approach based on the NADPH biosensor pSenSox and fluorescence-activated cell sorting (FACS) to search for variants of the NADPH-dependent ADH of *Lactobacillus brevis* (*Lb*ADH) with improved activity for the reduction of 2,5-hexanedione to (2*R*,5*R*)-hexanediol. In a library of approx. 1.4 × 10^6^ clones created by random mutagenesis we identified the variant *Lb*ADH^K71E^. Kinetic analysis of the purified enzyme revealed that *Lb*ADH^K71E^ had a ~ 16% lowered K_M_ value and a 17% higher V_max_ for 2,5-hexanedione compared to the wild-type *Lb*ADH. Higher activities were also observed for the alternative substrates acetophenone, acetylpyridine, 2-hexanone, 4-hydroxy-2-butanone, and methyl acetoacetate. K71 is solvent-exposed on the surface of *Lb*ADH and not located within or close to the active site. Therefore, K71 is not an obvious target for rational protein engineering. The study demonstrates that high-throughput screening using the NADPH biosensor pSenSox represents a powerful method to find unexpected beneficial mutations in NADPH-dependent alcohol dehydrogenases that can be favorable in industrial biotransformations.

## Introduction

Chiral alcohols with high enantiomeric purity are important intermediates for the synthesis of optically active fine chemicals that are used for example to produce pharmaceuticals and agrochemicals (Ager et al. 1996; Liese et al. 2006; Breuer et al. 2004). Alcohol dehydrogenases (ADHs) are used for the synthesis of chiral alcohols under very mild reaction conditions due to their high catalytic efficiency and selectivity (Hall and Bommarius 2011; Zheng et al. 2017). A prominent example is the NADPH-dependent ADH from *Lactobacillus brevis* (*Lb*ADH), which catalyzes the stereoselective reduction of prochiral ketones to the corresponding, mostly (*R*)-configured secondary alcohols (Hummel 1999; Rodriguez et al. 2014). *Lb*ADH is an attractive candidate for biotransformations because it is a robust and versatile biocatalyst with high regio- and stereoselectivity, a broad substrate range, and the ability to convert sterically demanding substrates (Leuchs and Greiner 2011). Its preferred substrates are prochiral ketones such as acetophenone with almost invariably a small methyl group as one substituent and a bulky (often aromatic) moiety (such as phenyl) as the other (Schlieben et al. 2005). The efficiency of substrate conversion by *Lb*ADH is influenced by the substrate size, the steric and electronic effects of the substrate as well as the thermodynamic stability of the products (Rodriguez et al. 2014). *Lb*ADH is active as a homotetramer with 251 amino acid residues and a molecular mass of 26.6 kDa per subunit (Riebel 1997; Niefind et al. 2003). It is a short-chain Mg^2+^-dependent reductase that uses the NADPH as cofactor. The crystal structure of *Lb*ADH has been solved (Niefind et al. 2003; Schlieben et al. 2005). The non-covalently bound cofactor NADPH is essential for catalysis and must be recycled efficiently to make the biotransformation economically feasible (Leuchs and Greiner 2011; Döbber et al. 2018).

Because of the industrial relevance of ADHs, their improvement for various applications is of high interest, such as the optimization of specificity or catalytic activity or the broadening of the substrate spectrum (Hall and Bommarius 2011). The approaches used for obtaining improved ADH variants, such as directed evolution or rational design, have recently been reviewed (Zhang et al. 2015). In directed evolution, a large number of variants of a particular enzyme created by random and/or targeted mutagenesis is screened for the desired property (Farinas et al. 2001). However, the success of this approach is often restricted by the lack of an efficient high-throughput (HT) screening assay. Typically, screenings of mutant libraries involve dedicated assays for a certain substrate or product, because the majority of molecules of interest do not lead to an easily observable phenotype (Bloch 2006). In the case of ADHs, the consumption or production of NAD(P)H can be measured or colorimetric assays can be employed (Zhang et al. 2015), but this limits the number of variants that will be tested e.g. in 384-well microtiter plates in practice to 10^4^–10^5^. Fluorescence-activated cell sorting (FACS) allows screening of up to 80,000 single cells per second and thus enables HT-screening of 10^7^–10^9^ variants, if an ADH assay suitable for FACS is available (van Rossum et al. 2013).

Genetically encoded biosensors based on transcription factors controlling the synthesis of a fluorescent reporter protein are highly useful tools for HT-screening in strain and enzyme development (Dietrich et al. 2010; Eggeling et al. 2015; Mahr and Frunzke 2016; Rogers et al. 2016). We previously reported a transcription factor-based NADPH biosensor allowing HT-screening of NADPH-dependent enzymes via fluorescence-activated cell sorting (FACS) of an *Escherichia coli*-based mutant library (Siedler et al. 2014). The NADPH biosensor is encoded by the plasmid pSenSox and consists of the transcription factor SoxR, its target promoter P_*soxS*_, and the reporter gene *eyfp*. The SoxRS system of *E. coli* triggers the response to oxidative stress (Greenberg et al. 1990; Tsaneva and Weiss 1990) and SoxR was found to be activated, besides other stimuli, by a reduction of the NADPH/NADP^+^ ratio in the cell (Liochev and Fridovich 1992; Krapp et al. 2011). We recently confirmed that the pSenSox biosensor responds to various NADPH-related processes in *E. coli*, such as the presence of redox-cycling drugs, the absence of the SoxR-reducing proteins RsxABCDGE and RseC, and the absence of the transhydrogenases PntAB and/or SthA (Spielmann et al. 2018).

*Escherichia coli* cells carrying pSenSox become fluorescent during NADPH-dependent biotransformation processes due to a high rate of NADPH consumption. Using the reduction of methyl acetoacetate to *R*-methyl 3-hydroxybutyrate by *Lb*ADH as model reaction, it was demonstrated that the specific eYFP fluorescence of cells correlates both with the substrate concentration and, when the substrate concentration is kept constant, with the specific *Lb*ADH activity (Siedler et al. 2014). Due to the latter property, one promising application of the NADPH biosensor is the FACS-based HT-screening of libraries with a high number of variants of NADPH-dependent enzymes. A proof of concept approach led to the identification of an *Lb*ADH variant with a slightly increased activity, but reduced affinity for the substrate 4-methyl-2-pentanone (Siedler et al. 2014).

In the present study, we applied the pSenSox biosensor to screen an *Lb*ADH library in *E. coli* by FACS for variants that enable an improved biotransformation of 2,5-hexanedione to (2*R*,5*R*)-hexanediol. This compound serves as a building block for the synthesis of fine chemicals, pharmaceuticals, agrochemicals and chiral phosphine ligands (Haberland et al. 2002; Machielsen et al. 2009). We identified the variant *Lb*ADH^K71E^ and showed that it has an increased activity for the reduction of 2,5-hexanedione, but also various other substrates.

## Materials and methods

### Chemicals, bacterial strains, plasmids and growth conditions

Unless specified otherwise the chemicals were purchased from Sigma-Aldrich GmbH (Steinheim, Germany), BD Biosciences (Franklin Lakes, USA), or Carl Roth (Karlsruhe, Deutschland). All bacterial strains and plasmids used in this work are listed in Table 1. One Shot™ TOP10 Electrocomp *E. coli* cells (Invitrogen, Karlsruhe, Germany) were used for cloning and screening purposes. Transformation of *E. coli* cells was performed as described (Hanahan 1983). Cells were cultivated at 37 °C in liquid 2xTY medium consisting of 16 g L^−1^ tryptone (BD Biosciences, Franklin Lakes, USA), 10 g L^−1^ yeast extract, and 5 g L^−1^ sodium chloride, in terrific broth (TB) medium (12 g L^−1^ tryptone, 24 g L^−1^ yeast extract, 4 mL glycerol, 12.54 g L^−1^ K_2_HPO_4_, 2.31 g L^−1^ KH_2_PO_4_; pH 7.0), or on LB agar (Carl Roth, Karlsruhe, Deutschland). Plasmids were selected by adding carbenicillin to the medium to a final concentration of 100 µg mL^−1^. BD™ *FACSFlow* Sheath Fluid for flow cytometry applications was purchased from BD Biosciences (Franklin Lakes, USA).

**Table 1:** Bacterial strains and plasmids used in this study

Strain or plasmid	Relevant characteristics	Source or reference
*Escherichia coli*		
TOP10	*mcrA*, Δ(*mrr*-*hsd*RMS-*mcrBC*), Phi80*lacZ(del)M15*, Δ*lacX74*, *deoR*, *recA1*, *araD139*, Δ(*ara*-*leu*)7697, *galU*, *galK*, *rpsL(SmR)*, *endA1*, *nupG,* strain used for general cloning procedures	Invitrogen
C43(DE3)	F– *ompT gal dcm hsdSB*(*rB*- *mB*-)(DE3), strain used for protein expression	Miroux and Walker (1996)
Plasmids		
pSenSox	Amp^R^; pBtac-*Lbadh* derivative containing the *soxRS*-based NADPH biosensor and the *Lbadh*^WT^ gene under transcriptional control of the *tac* promoter	Siedler et al. (2014)
pSenNeg	Amp^R^; pSenSox derivative with an incomplete *Lbadh*^WT^ gene preventing synthesis of an active *Lb*Adh^WT^	Siedler et al. (2014)
pSenSox-*Lbadh*^K71E^	Amp^R^; pSenSox derivative with the *Lbadh*^K71E^ gene under control of the *tac*-promoter	This study
pASK-IBA5plus-*Lbadh*^WT^	Amp^R^; pASK-IBA5plus derivative for production of *Lb*Adh^WT^ with N-terminal Strep-tag II under control of the *tet*-promoter/operator	Prof. W. Kroutil, Department of Chemistry, University of Graz, Austria
pASK-IBA5plus-*Lbadh*^K71E^	Amp^R^; pASK-IBA5plus derivative carrying the gene construct for the purification of the mutant *Lb*Adh^K71E^ protein with an N-terminal Strep-Tactin affinity tag (Strep-tag II) under transcriptional control of the *tet*-promoter/operator	This study

### Recombinant DNA work and library construction

Standard methods such as PCR were carried out according to established protocols (Sambrook and Russell 2001). Oligonucleotides were synthesized by Eurofins Genomics (Ebersberg, Germany) and are listed in Table 2. All plasmids were sequenced by Eurofins Genomics (Ebersberg, Germany). For random mutagenesis of the *Lbadh*^WT^ gene, error-prone PCR was performed using the oligonucleotide pair pSenSox-*Lbadh*-fw and pSenSox-*Lbadh*-rv, the plasmid pSenSox as template, and the GeneMorph II Random Mutagenesis Kit (Agilent Technologies, Santa Clara CA, USA). The resulting mutated *Lbadh* fragments were cloned by Gibson assembly (Gibson et al. 2009) into a pSenSox fragment obtained by restriction with EcoRI and HindIII to remove the *Lbadh*^WT^ gene. The Gibson assembly mixture representing the *Lbadh*^Library^ was used to transform electrocompetent cells of *E. coli* TOP10. The resulting library was composed of about 1.4 × 10^6^ individual clones and was used for preparation of glycerol stocks.

**Table 2:** Oligonucleotides used in this study

Oligonucleotide	Sequence (5′ → 3′) and properties
pSenSox-*Lbadh*^−^fw	**CAATTTCACACAGGAAACAGGCGGCCGC**ATGTCTAACCGTTTGGATG
pSenSox-*Lbadh*^−^rv	**CTCTCATCCGCCAAAACAGAGAATTC**CTATTGAGCAGTGTAGCC
pSenSox-*Lbadh*^Library^ sequencing fw	TAATCATCGGCTCGTATAATGTGTG
pSenSox-*Lbadh*^Library^ sequencing rv	GCTTCTGCGTTCTGATTTAATCTG
*Lbadh*-mutagenesis A412G fw	GATGAAGATGGTTGGACCGAACTGTTTGATGCAACC
*Lbadh*-mutagenesis A412G rv	GGTTGCATCAAACAGTTCGGTCCAACCATCTTCATC
pASK-IBA5plus-*Lbadh*^K71E^ sequencing fw	GAAATAATTTTGTTTAACTTTAAGAAGG
pASK-IBA5plus-*Lbadh*^K71E^ sequencing rv	CCATTTTTCACTTCACAGGTCAAGC

Overlapping regions required for Gibson assembly in oligonucleotides used for cloning of the *Lbadh*^Library^ genes into pSenSox cut with EcoRI and HindIII are shown in bold

The plasmid pASK-IBA5plus-*Lbadh*^K71E^ was generated by site-directed mutagenesis with pASK-IBA5plus-*Lbadh*^WT^ as template, the oligonucleotide pair *Lbadh*-mutagenesis-A412G-fw and *Lbadh*-mutagenesis-A412G-rv, and the PfuUltra II Hotstart PCR Master Mix (Agilent Technologies, Santa Clara CA, USA). The plasmid pSenSox-*Lbadh*^K71E^ was obtained by amplifying the *Lbadh*^K71E^ gene with the oligonucleotides pSenSox-*Lbadh*^−^fw and pSenSox-*Lbadh*^−^rv and pASK-IBA5plus-*Lbadh*^K71E^ as template and cloning of the PCR product into plasmid pSenSox cut with HindIII and EcoRI at the former position of the *Lbadh*^WT^ gene.

### Fluorescence-activated cell sorting (FACS)

Flow cytometric analysis and cell sorting were performed with a FACS ARIA II high-speed cell sorter (BD Biosciences, Franklin Lakes, NJ, USA) and the BD FACSDiva™ software 6.1.3.

eYFP fluorescence of single cells was measured by using an excitation wavelength of 488 nm emitted by a blue solid-state laser and an emission wavelength of 533 ± 30 nm at a sample pressure of 70 psi. A threshold was set to exclude non-bacterial particles on the basis of forward scatter (FSC) area versus side scatter (SSC) area. The flow rate was set to analyze 2000–4000 cells per second. For graphical representation of the obtained data as dot plots or histograms, the software FlowJo (FlowJo, LLC, Ashland, OR, USA) was used.

The strategy used to isolate cells containing *Lb*ADH variants with improved activity for reduction of 2,5-hexanedione included three enrichments steps with positive selection followed by one negative selection step and another positive selection step. A 1-mL culture of *E. coli* TOP10/pSenSox-*Lbadh*^Library^ in 2xTY medium containing 100 µg mL^−1^ carbenicillin with an initial optical density at 600 nm (OD_600_) of 0.2 was incubated for 4 h at 37 °C and 900 rpm in a 48-well non-transparent Flowerplate (m2p-labs GmbH, Baesweiler, Deutschland). Then the cells were harvested by centrifugation and resuspended in fresh 2xTY medium supplemented with 100 µg mL^−1^ carbenicillin to a final OD_600_ of 3 or higher. 800 µL of these suspensions were transferred into another 48-well Flowerplate and after addition of 100 µL 2,5-hexanedione to a final concentration of 70 mM, the plate was incubated for 2.5 h at 37 °C and 900 rpm for NADPH-dependent reduction of 2,5-hexanedione to (2*R*,5*R*)-hexanediol and concomitant NADPH-dependent expression of *eyfp*. Then the cultures were diluted 50-fold in sterile-filtered BD™ FACSFlow Sheath Fluid (BD, Franklin Lakes, USA) and subjected to FACS.

The sorting gate was set to include the 1% most fluorescent cells. 3 × 10^4^ of these cells were sorted into fresh 2xTY medium containing 100 µg mL^−1^ carbenicillin. After overnight cultivation, a new 1-mL culture was inoculated, incubated for 4 h and then used again for biotransformation of 2,5-hexanedione. Afterwards, the cells were again screened by FACS, 3 × 10^4^ of the most fluorescent cells were isolated and the positive selection repeated for a third time. After culturing the cells of the third enrichment step, they were subjected to a mock biotransformation in which 2,5-hexanedione was omitted and then analyzed by FACS. In this case, the non-fluorescent cells were sorted in order to remove cells showing high fluorescence independent of *Lb*ADH-catalyzed 2,5-hexanedione reduction. For setting the sorting gate, cells of *E. coli* TOP10/pSenNeg were used, which lack an active *Lb*ADH. 1 × 10^5^ library cells within the negative sorting gate were collected in fresh 2xTY medium with 100 µg mL^−1^ carbenicillin. After overnight cultivation, a fourth round of positive selection was performed using cells after 2.5 h biotransformation of 2,5-hexanedione. Cells of *E. coli* TOP10/pSenSox with wild-type *Lb*ADH were used as reference and 240 library cells showing a higher fluorescence than the reference were spotted on LB agar plates with 100 µg mL^−1^ carbenicillin.

83 out of the 240 spotted cells formed colonies after overnight incubation at 37 °C and were subsequently analyzed in a BioLector microcultivation system (m2p-laps, Baesweiler, Germany) by following eYFP fluorescence and growth as described below. 65 clones showed the same fluorescence pattern with a higher fluorescence compared to the reference strain *E. coli* TOP10/pSenSox expressing the wild-type *Lbadh*^WT^ gene in the presence of the substrate 2,5-hexanedione. The plasmids of four of these strains were isolated and sequenced. All four clones carried a single G → A transition in the *Lbadh* gene resulting in the amino acid exchange K71E.

### Biotransformation and monitoring of the NADPH biosensor response

The fluorescence intensity of the NADPH biosensor signal was measured during the whole-cell biotransformation of the substrate 2,5-hexanedione. To test the difference in the fluorescence intensity, pre-cultures of the cultures obtained after FACS screening and the *E. coli* TOP10/pSenSox culture as positive control were incubated overnight at 37 °C and 130 rpm in 5 mL 2xTY medium containing 100 μg mL^−1^ carbenicillin. The pre-cultures were used to inoculate main cultures in 2xTY medium with 100 μg mL^−1^ carbenicillin to an OD_600_ of 0.05, which were cultivated at 37 °C and 130 rpm. The cells were further cultivated for 5 h, harvested by centrifugation (4 °C, 4713*g*, 15 min) and resuspended in 5 mL fresh 2xTY medium supplemented with 100 μg mL^−1^ carbenicillin to a final OD_600_ of 5. 800 µL of these suspensions were transferred into a 48-well Flowerplate (m2p-laps, Baesweiler, Germany). To start the biotransformation of the substrates, 100 µL of the substrate 2,5-hexanedione dissolved in ddH_2_O was added to the cultures to a final concentration of 70 mM. 100 µL ddH_2_O were added to the cultures instead of the substrates as negative controls. After the desired additions, the Flowerplates were incubated in a BioLector microcultivation system at 30 °C and 1200 rpm (shaking diameter 3 mm) and eYFP fluorescence (excitation wavelength 485 nm, emission wavelength of 520 nm) and cell density (as backscattered light at 620 nm) were monitored online (Kensy et al. 2009).

### *Lb*ADH overproduction and purification

For enzyme production, *E. coli* C43(DE3) carrying pASK-IBA5plus-*Lbadh*^WT^ or pASK-IBA5plus-*Lbadh*^K71E^ was cultivated in TB medium supplemented with 1 mM MgCl_2_ and 100 μg mL^−1^ carbenicillin. Pre-cultures were used to inoculate 1 L main cultures in a 5 L shaking flask to a starting OD_600_ of 0.1. The main cultures were shaken at 37 °C and 130 rpm until an OD_600_ of 0.6 was reached. Then *Lb*ADH overproduction was induced with 0.2 mg L^−1^ anhydrotetracycline and the cultures were then incubated for 20 h at 20 °C and 130 rpm. Subsequently, the cells were harvested by centrifugation (4 °C, 4713*g*, 30 min), resuspended in 5 mL lysis buffer (100 mM Tris/HCl buffer at pH 7.2 with 1 mM MgCl_2_, 1 µg mL^−1^ DNAse and protease inhibitor (cOmplete™ ULTRA Tablets, Mini, EDTA-free, *EASYpack* Protease Inhibitor Cocktail, Roche, Basel, Switzerland), and incubated for 20 min on ice. For cell disruption, the cell suspension was passed three times through a French pressure cell at 110 MPa. To sediment intact cells and cell debris, the extract was centrifuged for 60 min at 10,000*g* and 4 °C. The resulting supernatant was filtered through a 0.22 µm filter (Millex-GP, polyethersulfon, Merck Millipore, Tullagreen, Ireland) and used for a two-step purification process with an Äkta™ Pure chromatography system (GE Healthcare Bio-Sciences, Uppsala, Sweden). First, *Lb*ADH was isolated by affinity chromatography using a 1 mL Strep-Trap™ HP column (GE Healthcare Bio-Sciences, Uppsala, Sweden) equilibrated with 100 mM Tris/HCl buffer pH 7.2 containing 1 mM MgCl_2_ according to the protocol provided by the manufacturer. For elution of specifically bound proteins, equilibration buffer supplemented with 2.5 mM desthiobiotin was used. For the second purification step, size exclusion chromatography was performed using a Superdex™ 200 increase 10/300 GL column (GE Healthcare Bio-Sciences, Uppsala, Sweden) equilibrated with buffer A (50 mM triethanolamine hydrochloride (TEA) containing 1 mM MgCl_2_ and adjusted to pH 7.0 with 1 M NaOH). *Lb*ADH^WT^ and *Lb*ADH^K71E^ were eluted with buffer A at a flow rate of 0.75 mL min^−1^. To determine the molecular mass of the eluted proteins, a calibration curve was established by performing size exclusion chromatography under the same conditions with proteins of known size, namely carbonic anhydrase (29 kDa), bovine serum albumin (66 kDa), alcohol dehydrogenase (150 kDa) and β-amylase (200 kDa). Protein concentrations were determined using a bicinchoninic acid assay (Interchim, Montluçon, France). 1 µg protein of the elution fraction obtained after affinity purification and 1 µg protein of the elution fraction obtained after size exclusion chromatography were analyzed by sodium dodecyl sulfate-polyacrylamide gel electrophoresis (SDS-PAGE) using a Mini-PROTEAN^®^ TGX™ Any kD™ gel (Bio-Rad Laboratories, Hercules CA, USA).

### Dynamic light scattering (DLS) and thermal shift assay

DLS analysis was carried out on the concentrated enzyme (0.6–1.2 mg mL^−1^) using a DynaPro Nanostar (Wyatt technology, Santa Barbara, CA, USA). The hydrodynamic radius was measured, and the molecular weight was calculated by DYNAMICS V6.

For the thermal shift assay, 20 µL of the protein solution (0.6–1.2 mg mL^−1^) was mixed with 5 µL 100 × SYPRO Orange in a plate suitable for RT-PCR and sealed with a transparent cover. In a CFX96 RT-PCR machine (Bio-Rad, Hercules, CA, USA), a melt curve program was set up where the temperature ramps from 20 to 95 °C with 0.5 °C increments after a 10 s delay. At each interval, a fluorescence measurement using the SYBR Green filter set was performed. The Bio-Rad software was used to determine the first derivative of the fluorescence signal and the temperature. The maximum of this curve was taken as the apparent *T*_*m*_ of the protein.

### Activity of *Lb*ADH^WT^ and *Lb*ADH^K71E^ with various substrates

The kinetic properties of purified *Lb*ADH^WT^ and *Lb*ADH^K71E^ were analyzed using an assay in which the oxidation of NADPH was followed at 340 nm using a JASCO V560 UV/VIS spectrophotometer (JASCO, Gross-Umstadt, Germany) equipped with a water bath-heated cell holder set at 30 °C. The assay was performed in disposable semi-micro cuvettes made of polystyrene (Sarstedt, Nürnbrecht, Germany) containing 1 mL assay buffer composed of 50 mM TEA pH 7.0, 1 mM MgCl_2_, 0.3 mM NADPH, 0.7 µg purified *Lb*ADH^WT^ or *Lb*ADH^K71E^, and different concentrations of the substrate. For 2,5-hexanedione, concentrations from 1 mM to 20 mM were used, for methyl acetoacetate 0.195 mM to 12.5 mM. To determine the K_M_ value for NADPH, 20 mM 2,5-hexanedione and NADHPH concentrations from 0.003 mM to 0.3 mM were used. The activity for acetophenone was determined at 5 mM, the activities for 2-acetylpyridine, 2-hexanone, and 4-hydroxy-2-butanone were determined at 10 mM. The progress of NADPH consumption was measured continuously for 1 min and used to calculate the corresponding enzyme activity using a molar extinction coefficient for NADPH at 340 nm of 6.22 mM^−1^ cm^−1^. The data were used to create Michaelis–Menten and Lineweaver–Burk plots. K_M_ and V_max_ values were determined by nonlinear regression of the Michaelis–Menten equation using the software GraphPad Prism 7 (GraphPad Software, La Jolla California USA).

### GenBank/EMBL accession numbers

The GenBank/EMBL accession number for the nucleotide sequence of the *Lbadh* gene is AJ544275. The GenBank/EMBL accession number for the amino acid sequence of the *Lb*ADH protein is CAD66648.

## Results

### Construction of an *Lb*ADH library and FACS screening

We aimed at finding *Lb*ADH variants with improved catalytic properties for the reduction of 2,5–hexandione to (2*R*,5*R*)-hexanediol by FACS-based HT-screening using the NADPH biosensor pSenSox. For this purpose, a library of *Lbadh* variants was generated by error-prone PCR and used to replace the *Lbadh*^WT^ gene of pSenSox. The resulting library pSenSox-*Lbadh*^Library^, present in *E. coli* TOP10, had an estimated size of 1.4 × 10^6^ clones. A culture expressing the *Lbadh*^Library^ was used for the biotransformation of 2,5-hexanedione by incubation for 2.5 h in 2xTY medium supplemented with 70 mM of the diketone. Preliminary studies had revealed that cells expressing *Lbadh*^WT^ showed maximal specific fluorescence during the biotransformation of 2,5-hexanedione after 2 to 3 h.

**Fig. 1:**
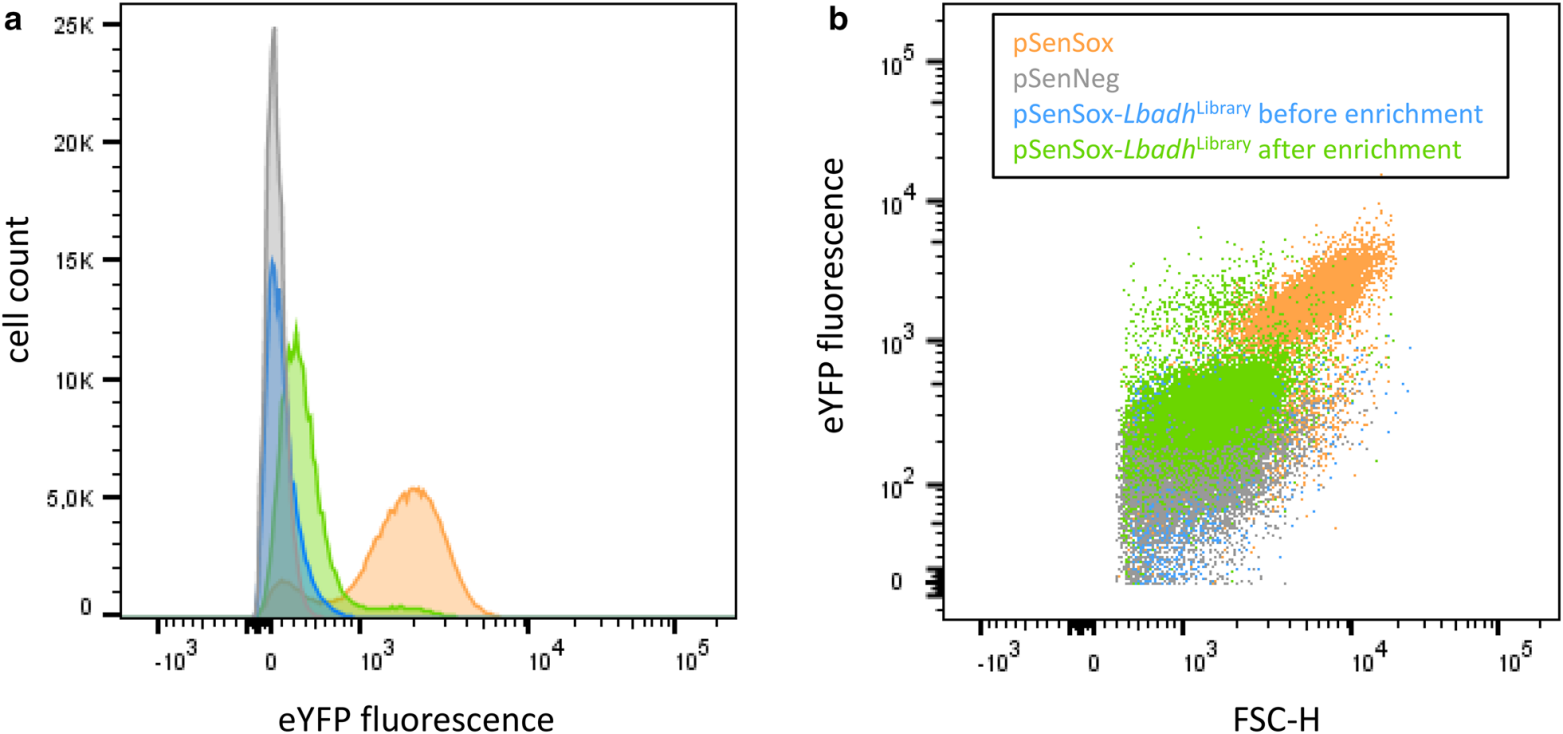
FACS analysis of the *Lb*ADH mutant library. Comparison of the fluorescence distribution of the *E. coli* TOP10/pSenSox-*Lbadh*^Library^ culture before (blue) and after three positive enrichment steps followed by one negative and another positive enrichment step (green). *E. coli* TOP10/pSenSox cells (orange) were used as positive control and *E. coli* TOP10/pSenNeg cells (gray) as negative control. Before FACS analysis, cells of the different cultures were incubated for 2.5 h with the substrate 2,5-hexanedione. Prior to sorting, 2.5 × 10^5^ cells of each culture were analyzed. The plots were generated with the BD DIVA 6.1.3 software. **a** Histogram of the four *E. coli* cultures described above. **b** Dot plots of the four cultures described above displaying the eYFP fluorescence signal against the forward scatter height (FSC-H) reflecting the size of the cells

After biotransformation, the library cells were diluted and subjected to FACS (Fig. 1) as described in detail in the methods section. The entire screening process comprised three positive selection steps for cells showing high fluorescence after biotransformation of 2,5-hexanedione followed by a negative selection step. In the latter, cells showing high fluorescence independent of 2,5-hexanedione were excluded by collecting only those cells in the library that showed the same fluorescence as *E. coli* TOP10/pSenNeg cells lacking an active *Lb*ADH. After propagation, these cells were subjected to a fourth round of positive selection. 2.5 × 10^5^ cells of the library were analyzed and 240 cells showing a higher fluorescence than the reference strain *E. coli* TOP10/pSenSox containing wild-type *Lb*ADH were spotted on agar plates and incubated overnight at 37 °C. 83 cells (35%) formed colonies and were further analyzed.

### Initial characterization of isolated clones in the BioLector

To confirm the increased fluorescence of the 83 isolated clones, they were cultivated in a BioLector system in 2xTY medium with or without 70 mM 2,5-hexanedione. The specific fluorescence of the cultures (the ratio of absolute fluorescence over cell density determined as backscatter at 620 nm) was monitored online for around 24 h. As reference, the strain expressing the wild-type *Lbadh* gene was used. Several of the clones showed an increased specific fluorescence in the presence of 2,5-hexanedione compared to the absence of 2,5-hexanedione and a higher specific fluorescence than the reference strain with wild-type *Lb*ADH. Sequencing of the plasmids isolated from four of these clones revealed that all carried a single G → A transition in the *Lbadh* gene resulting in the amino acid exchange K71E. The finding that all four clones contained the same mutation is probably due the applied selection procedure involving four positive and one negative selection step.

To confirm that this mutation, rather than secondary mutations in the pSenSox plasmid or in the genomic DNA, was responsible for the increased fluorescence during the biotransformation of 2,5-hexanedione, the *Lbadh*^K71E^ gene was amplified by PCR, used to replace the wild-type *Lbadh* gene in plasmid pSenSox, and transferred into *E. coli* TOP10. Cultivation of the recombinant strain carrying pSenSox-*Lbadh*^K71E^ in a BioLector  system confirmed that it shows a higher specific fluorescence in the presence of 2,5-hexanedione than in the absence, and higher fluorescence than *E. coli* TOP10/pSenSox with wild-type *Lb*ADH (data not shown). This result supported the assumption that the K71E mutation in *Lb*ADH was responsible for the increased fluorescence and leads to improved properties for 2,5-hexanedione reduction.

### Purification and biochemical characterization of *Lb*ADH^WT^ and *Lb*ADH^K71E^

**Fig. 2:**
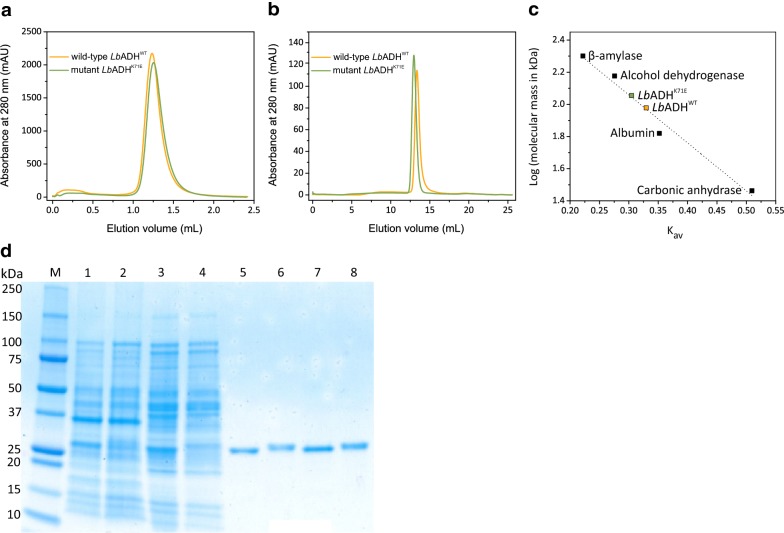
Purification of *Lb*ADH^WT^ and *Lb*ADH^K71E^. **a** Chromatogram of the affinity chromatography with the Strep-Trap™ HP column. **b** Chromatogram of the size-exclusion chromatography of the affinity-purified proteins with a Superdex™ 200 increase 10/300 GL column. **c** Calibration curve used for molecular mass determination obtained with proteins of known molecular mass: carbonic anhydrase (29 kDa), bovine serum albumin (66 kDa), alcohol dehydrogenase (150 kDa) and β-amylase (200 kDa). For molecular mass determination, the partition coefficient K_av_ was calculated and plotted against the logarithm of the molecular mass. *Lb*ADH^WT^ was shown to be tetrameric with a molecular mass of 107 kDa. *Lb*ADH^WT^ eluted with an apparent molecular mass of 95 kDa and *Lb*ADH^K71E^ with an apparent molecular mass of 113 kDa. **d** SDS-PAGE analysis of crude cell extracts (lanes 1 and 2) and soluble protein fractions (lanes 3 and 4) of *E. coli* C43(DE3)/pASK-IBA5plus-*Lbadh*^WT^ (lanes 1 and 3) and *E. coli* C43(DE3)/pASK-IBA5plus-*Lbadh*^K71E^ (lanes 2 and 4) and of purified *Lb*Adh^WT^ (lanes 5 and 7) and *Lb*Adh^K71E^ (lanes 6 and 8) after StrepTactin affinity chromatography (lanes 5 and 6) and after size-exclusion chromatography (lanes 7 and 8). The Mini-PROTEAN^®^ TGX™ any kD™ gel was stained with GelCode™ Blue Stain Reagent (Thermo Scientific, Rockford, USA). M, protein molecular mass standards

To characterize and compare the *Lb*ADH^K71E^ variant with the *Lb*ADH^WT^, both enzymes containing an N-terminal StrepTag-II were overproduced in *E. coli* C43(DE3) using the expression plasmids pASK-IBA5plus-*Lbadh*^WT^ and pASK-IBA5plus-*Lbadh*^K71E^ and purified by StrepTactin Sepharose affinity chromatography followed by size-exclusion chromatography (Fig. 2). The two proteins showed an identical elution profile in the affinity chromatography (Fig. 2a), but in the size-exclusion chromatography, the *Lb*ADH^K71E^ eluted slightly before *Lb*ADH^WT^ (Fig. 2b). Consequently, the apparent mass calculated from a calibration curve (Fig. 2c) derived from standard proteins was somewhat larger for *Lb*ADH^K71E^ (113 kDa) than for *Lb*ADH^WT^ (95 kDa). The elution volume difference of ~ 0.39 mL between *Lb*ADH^WT^ and *Lb*ADH^K71E^ was consistent for three independent protein preparations with varying sample order and indicated a structural change triggered by the K71E exchange. The apparent native masses of 95 kDa and 113 kDa are in agreement with the known tetrameric structure of *Lb*ADH (calculated mass of monomer including StrepTag-II is 28.2 kDa). A structural change caused by the K71E exchange became also apparent after analysis of the purified proteins by SDS-PAGE: *Lb*ADH^K71E^ shows a slightly higher apparent mass than *Lb*ADH^WT^ (Fig. 2d).

To further analyze the structural difference of the two *Lb*ADH variants, dynamic light scattering (DLS) was performed with the purified enzymes. The results showed a monodisperse sample with a slight amount of aggregates that were more prevalent for the wild-type ADH than for the K71E variant. The radius and calculated mass (based on a globular shape) were slightly smaller for *Lb*ADH^K71E^ (r = 3.75 ± 0.07 nm; 72.5 ± 3.5 kDa) than for *Lb*ADH^WT^ (r = 3.85 ± 0.07 nm; 78.5 ± 4.9 kDa), but the difference was too small to be significant.

To test whether the K71E exchange has an influence on *Lb*ADH stability, the apparent melting point of the two protein variants was determined using a thermal shift assay with the dye SYPRO orange. *Lb*ADH^K71E^ appeared to be slightly more stable with a melting point of 52.8 ± 0.3 °C in comparison to *Lb*ADH^WT^ with a melting point of 51.5 ± 0.0 °C (data of three technical replicates).

**Fig. 3:**
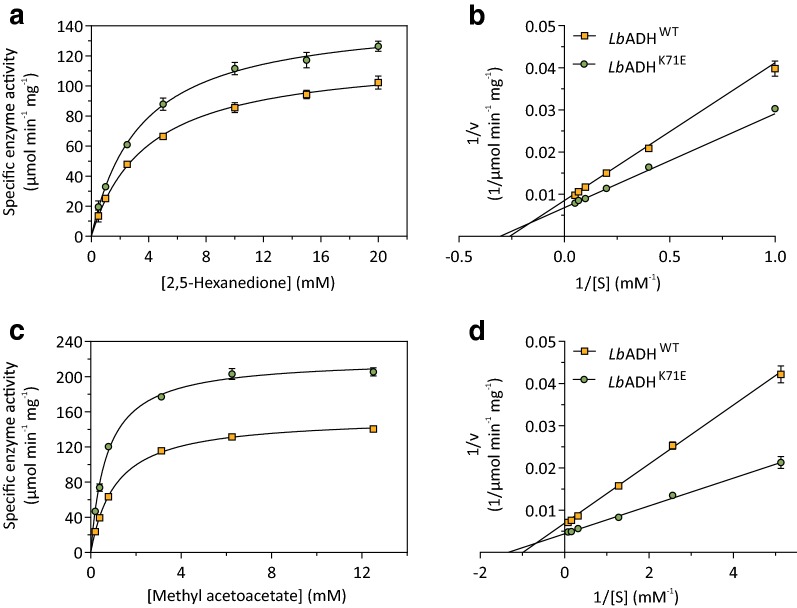
Representative Michaelis–Menten plots (**a**, **c**) and Lineweaver–Burk plots (**b**, **d**) for NADPH-dependent reduction of 2,5-hexanedione to (2*R*,5*R*)-hexandiol (**a**, **b**) and of methyl acetoacetate to (*R*)-methyl 3-hydroxybutyrate (**c**, **d**) by purified *Lb*ADH^WT^ and *Lb*ADH^K71E^. Data are mean values from three technical replicates of a single protein preparation

The pure enzymes were used for kinetic analysis using a spectrophotometric assay measuring NADPH consumption at 340 nm (see Methods section for details). In initial studies, the affinity and activity for the substrate 2,5-hexanedione were determined using Michaelis–Menten and Lineweaver–Burk plots (Fig. 3, Table 3). The K_M_ value determined for *Lb*ADH^K71E^ (4.3 ± 0.5 mM) was 16% lower than the one determined for *Lb*ADH^WT^ (5.1 ± 0.6 mM). The maximal activity calculated for *Lb*ADH^K71E^ (173.3 ± 11.1 µmol min^−1^ mg^−1^) was 17% higher than that of *Lb*ADH^WT^ (148.5 ± 12.3 µmol min^−1^ mg^−1^). Consequently, *Lb*ADH^K71E^ is more active than *Lb*ADH^WT^ regarding the NADPH-depedent reduction of 2,5-hexanedione and has a higher affinity for this substrate. No activity was found when NADPH was replaced by NADH. To test whether the improved kinetic properties of *Lb*ADH^K71E^ are specific for 2,5-hexanedione, we determined the K_M_ and V_max_ values for methyl acetoacetate. Also for this substrate, *Lb*ADH^K71E^ showed a better affinity and a higher maximal activity than *Lb*ADH^WT^ (Fig. 3c, d, Table 3). In a further set of experiments, the activity of the two enzymes for four other substrates was compared at a single concentration. As summarized in Table 4, *Lb*ADH^K71E^ showed 21–39% higher activity for 2-acetylpyridine, 2-hexanone, acetophenone, and 4-hydroxy-2-butanone, suggesting that the positive effect of the K71E exchange on *Lb*ADH activity was independent of the substrate.

**Table 3:** Kinetic parameters of purifed *Lb*ADH^WT^ and *Lb*ADH^K71E^ for various substrates

Substrate	V_max_ (µmol min^−1^ mg^−1^)		K_M_ (mM)	
	*Lb*ADH^WT^	*Lb*ADH^K71E^	*Lb*ADH^WT^	*Lb*ADH^K71E^
2,5-Hexanedione^a^	148.5 ± 12.3	173.3 ± 11.1	5.1 ± 0.6	4.3 ± 0.5
Methyl acetoacetate^b^	154.2 ± 2.2	221.2 ± 3.3	1.13 ± 0.06	0.67 ± 0.04

^a^Mean values and standard deviation based on three separate protein preparations with three technical replicates per preparation

^b^Mean values and standard deviation based on a single protein preparation and three technical replicates

**Table 4:** Analysis of *Lb*ADH^WT^ and *Lb*ADH^K71E^ activity for the substrates methyl acetoacetate, 2-acetylpyridine, 4-hydroxy-2-butanone, acetophenone, and 2-hexanone

Substrate	Substrate concentration (mM)	*Lb*ADH^WT^ activity (µmol min^−1^ mg^−1^)	*Lb*ADH^K71E^ activity (µmol min^−1^ mg^−1^)	% increase
2-Acetylpyridine	10	104.1 ± 1.7	125.5 ± 1.1	21
2-Hexanone	10	22.8 ± 0.7	27.9 ± 1.1	22
acetophenone	5	29.8 ± 0.7	39.5 ± 0.7	33
4-Hydroxy-2-butanone	10	39.5 ± 1.3	54.9 ± 4.6	39

Mean values obtained from a single protein preparation and three technical replicates are shown

## Discussion

**Fig. 4:**
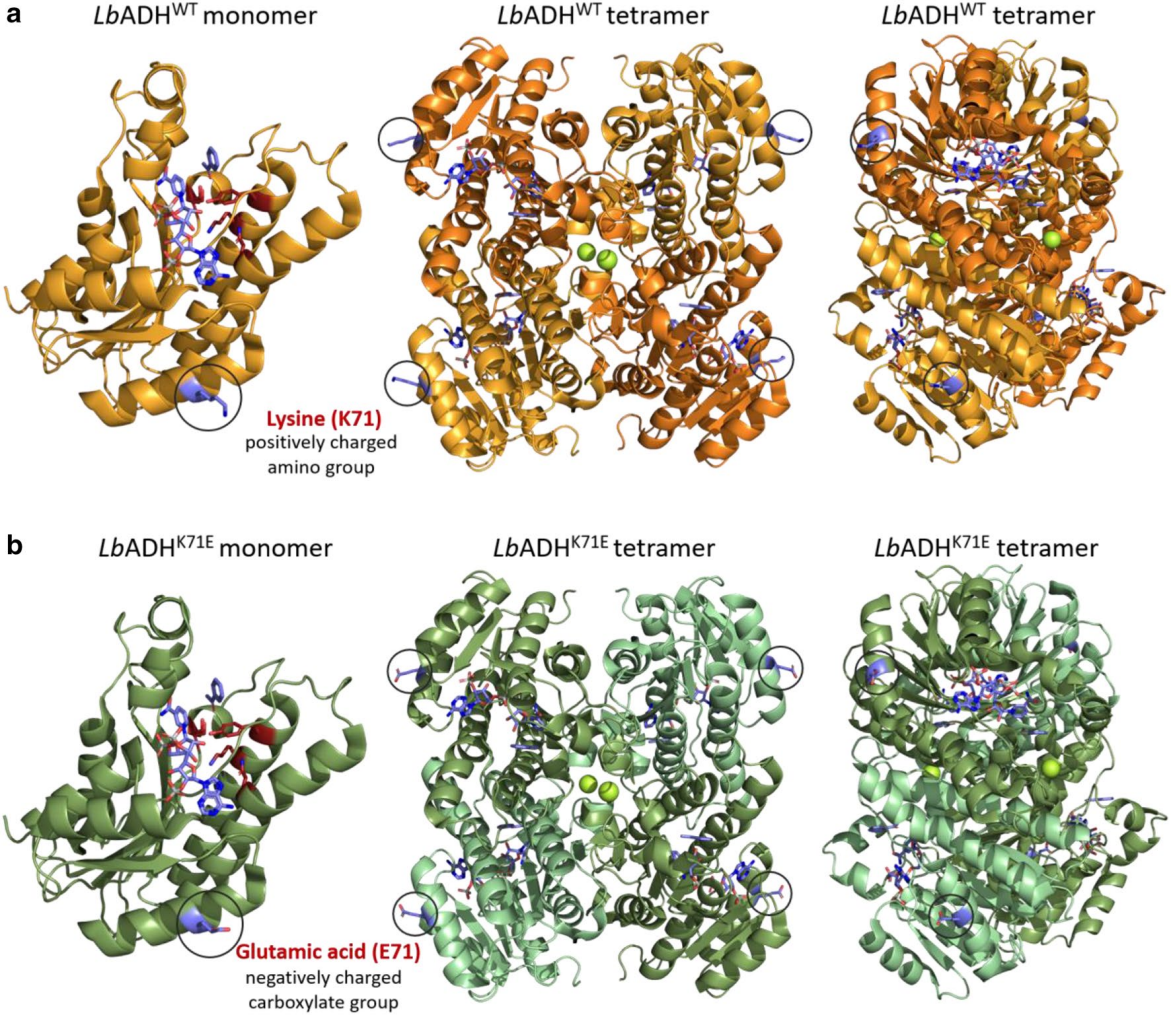
Crystal structure of the *Lb*ADH^WT^ monomer and the homotetramer in complex with the substrate acetophenone and the cofactor NADPH (Schlieben et al. 2005) (**a**) and modelled structure of *Lb*ADH^K71E^ (**b**). In the tetrameric structure, the Mg^2+^ ions needed for structure stabilization are shown as light green spheres. The amino acids at position 71 are shown as sticks and marked with a black circle. The residues Asn113, Ser142, Tyr155, Lys159 involved in the catalytic mechanism are shown in red as sticks in the monomer. The images were generated with the software PyMOL 2.2.0 (PDB code 1ZK4)

A FACS-based HT approach to identify optimized variants of *Lb*ADH for NADPH-dependent reduction of 2,5-hexanedione resulted in the isolation of *Lb*ADH^K71E^. The exchange of a lysine residue to a glutamic acid residue at position 71 led to an increased activity and a better affinity for 2,5-hexanedione, but also for the substrate methyl acetoacetate. Furthermore, up to 39% increased activities were found for the substrates 2-acetylpyridine, 2-hexanone, acetophenone, and 4-hydroxy-2-butanone. The crystal structure of the *Lb*ADH^WT^ homotetramer in complex with the substrate acetophenone, the cofactor NADPH and Mg^2+^ is available (Schlieben et al. 2005). Interestingly, position 71 is not located close to the active center at the protein core, but solvent-exposed on the protein surface (Fig. 4). According to PyMOL 2.2.0 (Schrödinger 2015), the distance of position 71 to the substrate acetophenone is around 28.0 Å and to the cofactor NADPH approximately 14.1 Å. The distance to the amino acids involved in catalysis, Asn113, Ser142, Tyr155, and Lys159 (Niefind et al. 2003) of the closest active site, is 19.4 Å, 27.4 Å, 27.1 Å and 25.7 Å, respectively. The distance to the active center suggests that the amino acid at position 71 is not directly involved in substrate binding and reduction.

Apart from the position of the mutation, the nature of the amino acid exchange has to be considered. The mutation led to an exchange of a lysine residue with a positively charged amino group by a glutamate residue with a negatively charged carboxyl group. The enzyme activity assays were performed at pH 7. The K71E exchange on the *Lb*AHD protein surface will influence its net charge and isoelectric point (pI), as these parameters depend on the content of ionizable groups and their p*K*_a_ values (Shaw et al. 2001). According to a protein parameter calculator (https://web.expasy.org/protparam), the K71E exchange resulted in a change of the pI from 5.44 for *Lb*Adh^WT^ to 5.17 for *Lb*Adh^K71E^. SDS-PAGE also suggested that the mutation K71E changed the electrostatic properties of the enzyme, because *Lb*ADH^K71E^ showed slower migration on the gel than *Lb*ADH^WT^ (Fig. 2d). In SDS-PAGE, proteins with a lower pI might migrate slower due to stronger negative charge repulsion with SDS (Shirai et al. 2008).

The net charge and the ionization state of individual residues affect many aspects of protein behavior including protein structure, stability, solubility, and function (Shaw et al. 2001; Pace et al. 2009). The slightly different elution profiles of *Lb*ADH^K71E^ and *Lb*ADH^WT^ in the size-exclusion chromatography (Fig. 2b) support a structural change caused by the K71E mutation. Moreover, electrostatic effects dominate hydration, denaturation, protein assembly, allostery and salt bridges, thermal stability and enzyme catalysis (Perutz 1978; Matthew 1985). Many enzyme reactions proceed via charged transition states and therefore stabilization of charges can be a major catalytic factor (Russell and Fersht 1987). In the case of *Lb*ADH, Tyr155 is assumed to serve as a catalytic acid, which is deprotonated in the course of reduction and requires stabilization by the positively charged Lys159 for this purpose (Schlieben et al. 2005; Jörnvall et al. 1995). The K71E exchange might have a positive influence on enzyme catalysis by stabilizing the charged transition state. Charged residues on the surface of proteins can also generate electrical potentials that extend many angstroms out into solution, enhancing substrate association rates and catalytic rates in enzymes (Sharp and Honig 1990).

It is surprising that a single mutation on the protein surface, as is the case of the *Lb*ADH^K71E^ variant, is sufficient to increase enzyme activity. Several studies have shown that it is possible to increase protein stability by improving electrostatic interactions among charged groups on the surface of folded proteins through multiple and even single mutations (Akke and Forsen 1990; Grimsley et al. 1999; Strickler et al. 2006; Gribenko et al. 2009). The main factors affecting stability are the relative free energies of the folded (G_f_) and the unfolded (G_u_) states. Protein stability is defined as the difference in Gibbs free energy, ∆G_u_, between the folded and the unfolded state. Specific algorithms have been developed to predict the effects of mutations on protein stability by estimating the changes in the difference in Gibbs free energy (∆∆G) between the wild-type and the mutated enzyme. The mutated enzyme is more stable if ∆∆G is positive. For the proteins *Lb*ADH^WT^ and *Lb*ADH^K71E^ the servers SDM (Pandurangan et al. 2017) and I-Mutant 2.0 (Capriotti et al. 2005) both calculated ∆∆G to be +3.51 kJ/mol. The program iPTREE-STAB (Huang et al. 2007) predicted that the mutation K71E has a stabilizing effect without giving a concrete value for ∆∆G. These predictions were supported by thermal shift assays revealing a slightly higher melting temperature for *Lb*ADH^K71E^ than for *Lb*ADH^WT^.

In summary, this study confirms the suitability of pSenSox for FACS-based high-throughput screening of ADH libraries to identify variants with improved catalytic properties. Although it is still unclear how the K71E exchange positively influences the K_M_ and v_max_ values, the improved catalytic and stability properties the *Lb*ADH^K71E^ variant should be favorable for biotechnological applications.

## Data Availability

The strains and plasmids described in this article are available from the corresponding author upon request.

## Abbreviations

FACS: fluorescence-activated cell sorting
*Lb*ADH: alcohol dehydrogenase of *Lactobacillus brevis*
SDS-PAGE: sodium dodecyl sulfate-polyacrylamide gel electrophoresis

## References

[CR1] Ager DJ, Prakash I, Schaad DR (1996). 1,2-Amino alcohols and their heterocyclic derivatives as chiral auxiliaries in asymmetric synthesis. Chem Rev.

[CR2] Akke M, Forsen S (1990). Protein stability and electrostatic interactions between solvent exposed charged side chains. Proteins.

[CR3] Bloch W (2006). Enzyme assays. High-throughput screening, genetic selection and fingerprinting. Anal Bioanal Chem.

[CR4] Breuer M, Ditrich K, Habicher T, Hauer B, Kesseler M, Sturmer R, Zelinski T (2004). Industrial methods for the production of optically active intermediates. Angew Chem Int Ed.

[CR5] Capriotti E, Fariselli P, Casadio R (2005). I-Mutant2.0: predicting stability changes upon mutation from the protein sequence or structure. Nucleic Acids Res.

[CR6] Dietrich JA, McKee AE, Keasling JD (2010). High-throughput metabolic engineering: advances in small-molecule screening and selection. Annu Rev Biochem.

[CR7] Döbber J, Pohl M, Ley SV, Musio B (2018). Rapid, selective and stable HaloTag-*Lb*ADH immobilization directly from crude cell extract for the continuous biocatalytic production of chiral alcohols and epoxides. React Chem Eng.

[CR8] Eggeling L, Bott M, Marienhagen J (2015). Novel screening methods—biosensors. Curr Opin Biotechnol.

[CR9] Farinas ET, Bulter T, Arnold FH (2001). Directed enzyme evolution. Curr Opin Biotechnol.

[CR10] Gibson DG, Young L, Chuang R-Y, Venter JC, Hutchison CA, Smith HO (2009). Enzymatic assembly of DNA molecules up to several hundred kilobases. Nat Methods.

[CR11] Greenberg JT, Monach P, Chou JH, Josephy PD, Demple B (1990). Positive control of a global antioxidant defense regulon activated by superoxide-generating agents in *Escherichia coli*. Proc Natl Acad Sci USA.

[CR12] Gribenko AV, Patel MM, Liu J, McCallum SA, Wang C, Makhatadze GI (2009). Rational stabilization of enzymes by computational redesign of surface charge-charge interactions. Proc Natl Acad Sci USA.

[CR13] Grimsley GR, Shaw KL, Fee LR, Alston RW, Huyghues-Despointes BM, Thurlkill RL, Scholtz JM, Pace CN (1999). Increasing protein stability by altering long-range coulombic interactions. Protein Sci.

[CR15] Haberland J, Hummel W, Daussmann T, Liese A (2002). New continuous production process for enantiopure (2*R*,5*R*)-hexanediol. Org Process Res Dev.

[CR16] Hall M, Bommarius AS (2011). Enantioenriched compounds via enzyme-catalyzed redox reactions. Chem Rev.

[CR17] Hanahan D (1983). Studies on transformation of *Escherichia coli* with plasmids. J Mol Biol.

[CR18] Huang LT, Gromiha MM, Ho SY (2007). iPTREE-STAB: interpretable decision tree based method for predicting protein stability changes upon mutations. Bioinformatics.

[CR19] Hummel W (1999). Large-scale applications of NAD(P)-dependent oxidoreductases: recent developments. Trends Biotechnol.

[CR20] Jörnvall H, Persson B, Krook M, Atrian S, Gonzalez-Duarte R, Jeffery J, Ghosh D (1995). Short-chain dehydrogenases/reductases (SDR). Biochemistry.

[CR21] Kensy F, Zang E, Faulhammer C, Tan RK, Büchs J (2009). Validation of a high-throughput fermentation system based on online monitoring of biomass and fluorescence in continuously shaken microtiter plates. Microb Cell Fact.

[CR22] Krapp AR, Humbert MV, Carrillo N (2011). The *soxRS* response of *Escherichia coli* can be induced in the absence of oxidative stress and oxygen by modulation of NADPH content. Microbiology.

[CR23] Leuchs S, Greiner L (2011). Alcohol dehydrogenase from *Lactobacillus brevis*: a versatile robust catalyst for enantioselective transformations. Chem Biochem Eng.

[CR24] Liese A, Seelbach K, Wandrey C (2006). Industrial biotransformation.

[CR25] Liochev SI, Fridovich I (1992). Fumarase C, the stable fumarase of *Escherichia coli*, is controlled by the *soxRS* regulon. Proc Natl Acad Sci USA.

[CR26] Machielsen R, Looger LL, Raedts J, Dijkhuizen S, Hummel W, Hennemann H-G, Daussmann T, van der Oost J (2009). Cofactor engineering of *Lactobacillus brevis* alcohol dehydrogenase by computational design. Eng Life Sci.

[CR27] Mahr R, Frunzke J (2016). Transcription factor-based biosensors in biotechnology: current state and future prospects. Appl Microbiol Biotechnol.

[CR28] Matthew JB (1985). Electrostatic effects in proteins. Annu Rev Biophys Biophys Chem.

[CR29] Miroux B, Walker JE (1996). Over-production of proteins in *Escherichia coli*: mutant hosts that allow synthesis of some membrane proteins and globular proteins at high levels. J Mol Biol.

[CR30] Niefind K, Müller J, Riebel B, Hummel W, Schomburg D (2003). The crystal structure of *R*-specific alcohol dehydrogenase from *Lactobacillus brevis* suggests the structural basis of its metal dependency. J Mol Biol.

[CR31] Pace CN, Grimsley GR, Scholtz JM (2009). Protein ionizable groups: pK values and their contribution to protein stability and solubility. J Biol Chem.

[CR32] Pandurangan AP, Ochoa-Montano B, Ascher DB, Blundell TL (2017). SDM: a server for predicting effects of mutations on protein stability. Nucleic Acids Res.

[CR33] Perutz MF (1978). Electrostatic effects in proteins. Science.

[CR34] Riebel, B. (1997) Biochemische und molekularbiologische Charakterisierung neuer mikrobieller NAD(P)-abhängiger Alkoholdehydrogenasen. Dissertation, Heinrich-Heine-Universität Düsseldorf, Germany

[CR35] Rodriguez C, Borzecka W, Sattler JH, Kroutil W, Lavandera I, Gotor V (2014). Steric vs. electronic effects in the *Lactobacillus brevis* ADH-catalyzed bioreduction of ketones. Org Biomol Chem.

[CR36] Rogers JK, Taylor ND, Church GM (2016). Biosensor-based engineering of biosynthetic pathways. Curr Opin Biotechnol.

[CR37] Russell AJ, Fersht AR (1987). Rational modification of enzyme catalysis by engineering surface charge. Nature.

[CR38] Sambrook J, Russell D (2001). Molecular cloning. A laboratory manual.

[CR39] Schlieben NH, Niefind K, Müller J, Riebel B, Hummel W, Schomburg D (2005). Atomic resolution structures of *R*-specific alcohol dehydrogenase from *Lactobacillus brevis* provide the structural bases of its substrate and cosubstrate specificity. J Mol Biol.

[CR40] Schrödinger LLC (2015). The PyMOL molecular graphics system. Version.

[CR41] Sharp KA, Honig B (1990). Electrostatic interactions in macromolecules: theory and applications. Annu Rev Biophys Biophys Chem.

[CR42] Shaw KL, Grimsley GR, Yakovlev GI, Makarov AA, Pace CN (2001). The effect of net charge on the solubility, activity, and stability of ribonuclease Sa. Protein Sci.

[CR43] Shirai A, Matsuyama A, Yashiroda Y, Hashimoto A, Kawamura Y, Arai R, Komatsu Y, Horinouchi S, Yoshida M (2008). Global analysis of gel mobility of proteins and its use in target identification. J Biol Chem.

[CR44] Siedler S, Schendzielorz G, Binder S, Eggeling L, Bringer S, Bott M (2014). SoxR as a single-cell biosensor for NADPH-consuming enzymes in *Escherichia coli*. ACS Synth Biol.

[CR45] Spielmann A, Baumgart M, Bott M (2019). NADPH-related processes studied with a SoxR-based biosensor in *Escherichia coli*. MicrobiologyOpen.

[CR46] Strickler SS, Gribenko AV, Gribenko AV, Keiffer TR, Tomlinson J, Reihle T, Loladze VV, Makhatadze GI (2006). Protein stability and surface electrostatics: a charged relationship. Biochemistry.

[CR47] Tsaneva IR, Weiss B (1990). *soxR*, a locus governing a superoxide response regulon in *Escherichia coli* K-12. J Bacteriol.

[CR48] van Rossum T, Kengen SW, van der Oost J (2013). Reporter-based screening and selection of enzymes. FEBS J.

[CR49] Zhang R, Xu Y, Xiao R (2015). Redesigning alcohol dehydrogenases/reductases for more efficient biosynthesis of enantiopure isomers. Biotechnol Adv.

[CR50] Zheng YG, Yin HH, Yu DF, Chen X, Tang XL, Zhang XJ, Yue-Y P, Wang YJ, Liu ZQ (2017). Recent advances in biotechnological applications of alcohol dehydrogenases. Appl Microbiol Biotechnol.

